# Fast and sensitive mapping of nanopore sequencing reads with GraphMap

**DOI:** 10.1038/ncomms11307

**Published:** 2016-04-15

**Authors:** Ivan Sović, Mile Šikić, Andreas Wilm, Shannon Nicole Fenlon, Swaine Chen, Niranjan Nagarajan

**Affiliations:** 1 Computational & Systems Biology, Genome Institute of Singapore, 60 Biopolis Street, #02-01 Genome, Singapore 138672, Singapore; 2 Centre for Informatics and Computing, Ruđer Bošković Institute, Bijenička 54, 10000 Zagreb, Croatia; 3 Faculty of Electrical Engineering and Computing, Department of Electronic Systems and Information Processing, University of Zagreb, Unska 3, 10000 Zagreb, Croatia; 4 Bioinformatics Institute, Singapore 138671, Singapore; 5 Faculty of Medicine and Institute for Life Sciences, University of Southampton, Southampton SO16 6YD, UK; 6 Division of Infectious Diseases, Department of Medicine, Yong Loo Lin School of Medicine, National University of Singapore, Singapore 119074, Singapore

## Abstract

Realizing the democratic promise of nanopore sequencing requires the development of new bioinformatics approaches to deal with its specific error characteristics. Here we present GraphMap, a mapping algorithm designed to analyse nanopore sequencing reads, which progressively refines candidate alignments to robustly handle potentially high-error rates and a fast graph traversal to align long reads with speed and high precision (>95%). Evaluation on MinION sequencing data sets against short- and long-read mappers indicates that GraphMap increases mapping sensitivity by 10–80% and maps >95% of bases. GraphMap alignments enabled single-nucleotide variant calling on the human genome with increased sensitivity (15%) over the next best mapper, precise detection of structural variants from length 100 bp to 4 kbp, and species and strain-specific identification of pathogens using MinION reads. GraphMap is available open source under the MIT license at https://github.com/isovic/graphmap.

## Abstract (web summary)

Read mapping and alignment tools are critical for many applications based on MinION sequencers. Here, the authors present GraphMap, a mapping algorithm designed to analyze nanopore sequencing reads, that progressively refines candidate alignments to handle potentially high error rates to align long reads.

## Graphical abstract

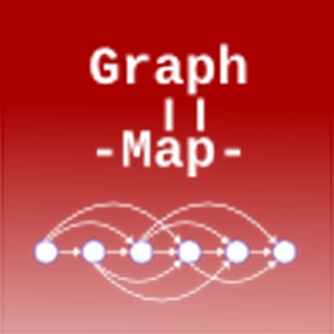

The release of Oxford Nanopore Technologies (ONT) MinION sequencers in 2014 ushered in a new era of cheap and portable long-read sequencers. Nanopore sequencers have transformative potential for research, diagnostic and low-resource applications. While some initial nanopore sequencing based applications have been reported (for example, scaffolding and resolution of repeats in genomes^1^ and variant detection in clonal haploid samples^2^), many others remain to be explored. In particular, diploid and rare-variant calling^3^, *de novo* genome assembly^4^, metagenome assembly and pathogen identification are all promising applications that will likely require new development of *in silico* techniques.

Read mapping and alignment tools are critical building blocks for many such applications. For mapping, reads from nanopore sequencing are particularly challenging due to their higher and non-uniform error profiles^5^. For example, one-demensional (1D) reads from the MinION sequencer have raw base accuracy <65–75%; higher quality two-dimensional (2D) reads (80–88% accuracy) comprise a fraction of all 2D reads and the total data set, with overall median accuracy being between 70 and 85% (refs 1, 6, 7, 8, 9). Reads from other short read (for example, Illumina; <1%) and long read (for example, PacBio; ∼10%) sequencing technologies have lower overall and mismatch (<1%) error rates. The increased read lengths in nanopore sequencing should facilitate mapping, reducing the ambiguity in location that is the major challenge for short read mappers. However, with current mappers, high-error rates result in a large fraction of reads and bases (10–30%) remaining unmapped or unused (for example, 1D reads) for downstream applications^1^,^6^,^7^. This is further compounded when comparing two error-prone reads to each other or mapping to an imperfect or distant reference. Thus, retaining sensitivity while accommodating high error or divergence rates is the key difficulty for current mapping methods. MinION error rates and profiles (that is, ratio of insertions, deletions and substitutions) can vary across chemistries, sequencing runs, read types and even within a read. Furthermore, other nanopore and single-molecule sequencing technologies may present a different distribution of error rates and profiles. Therefore, a general solution to mapping that is applicable to different error characteristics would have high utility for both current and future applications.

While alignment algorithms have been widely studied, gold-standard solutions such as dynamic programming (or even fast approximations such as BLAST) are too slow in practice for aligning high-throughput sequencing reads. To address this need, a range of read mapping tools have been developed that exploit the characteristics of second-generation sequencing reads (relatively short and accurate) by trading-off a bit of sensitivity for dramatic gains in speed^10^,^11^. The design decisions employed in these mappers are often tuned for specific error characteristics of a sequencing technology, potentially limiting their utility across technologies and error profiles. The less than ideal results reported in early studies using MinION data^12^ could therefore be in part due to the use of mappers (for example, BWA-MEM (ref. 6), BLASR (ref. 13) or LAST (ref. 14)) that are not suited to its error characteristics.

In this work, we present GraphMap, the first mapping algorithm designed for high sensitivity with current nanopore sequencing data. In solving the mapping problem for the potentially variable error profile of ONT MinION sequencers, GraphMap furthermore generally accommodates variable error characteristics, without the need for parameter tuning, while retaining high sensitivity and precision. Therefore, GraphMap allows uniform mapping of sequencing reads from disparate technologies (for example, Illumina, PacBio or ONT) with BLAST-like sensitivity and improved runtime. Experiments with several real and synthetic data sets demonstrate that GraphMap is a more sensitive mapper than BWA-MEM, DALIGNER, BLASR and LAST, while reporting accurate alignments with nanopore sequencing data. This benefits all downstream applications of mapping, as highlighted here with a few natural proof-of-concept applications for a low cost, long read, portable sequencer, that is, single-nucleotide polymorphism calling in complex regions of the human genome, structural variants (SVs; insertions and deletions) detection and real-time pathogen identification.

## Results

### Overview of the GraphMap algorithm

**Figure 1:**
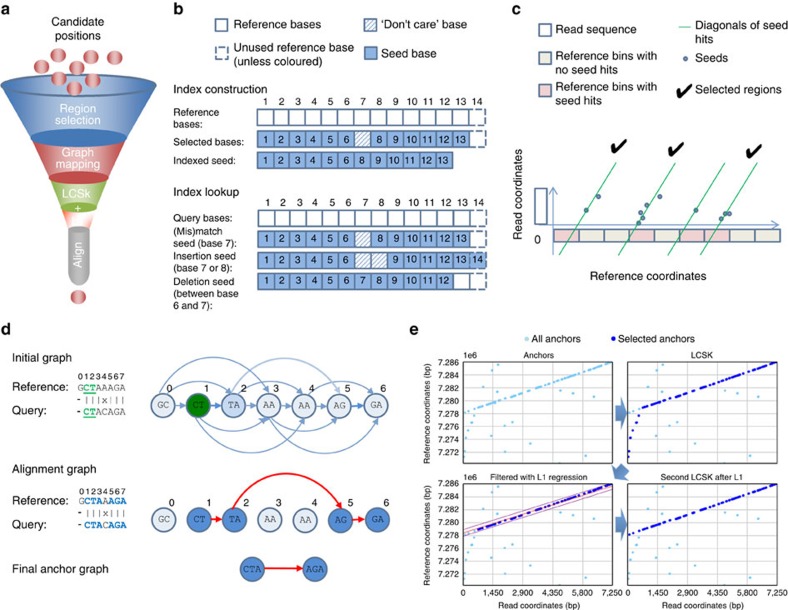
A schematic representation of stages in GraphMap. (**a**) Order of stages in the ‘read-funneling' approach used in GraphMap to refine alignments and reduce the number of candidate locations to one. (**b**) Structure of spaced seeds used for index construction and index look-up. For each position in the reference one seed is inserted into the index and for each position in the query, three seeds are looked up corresponding to the different error scenarios (**c**) Region selection by clustering of candidate seeds on the reference. Diagonals with sufficient number of seed hits are used to identify regions for further processing. (**d**) Generation of alignment anchors through a fast graph based ordering of seeds (*Graph Mapping*). After construction of the initial graph based on the reference, seeds from the query (2mers here; starting from the green seed) are looked up, and information in the graph propagated, to construct a maximal walk that serves as an anchor. (**e**) Filtering of seed matches using LCSk search and L1 regression. Anchors are chained into a monotonically increasing sequence, with outliers trimmed using L1 regression, to get an approximate alignment that helps select the correct mapping location.

The GraphMap algorithm is structured to achieve high-sensitivity and speed using a five-stage ‘read-funneling' approach as depicted in Fig. 1a. The underlying design principle is to have efficiently computable stages that conservatively reduce the set of candidate locations based on progressively defined forms of the read-to-reference alignment. For example, in stage I, GraphMap uses a novel adaptation of gapped spaced seeds^15^ to efficiently reduce the search space (Fig. 1b) and then clusters seed hits as a form of coarse alignment (Fig. 1c). These are then refined in stage II using graph-based vertex-centric processing of seeds to efficiently (allowing seed-level parallelism) construct alignment anchors (Fig. 1d). GraphMap then chains anchors using a kmer version of longest common subsequence construction (stage III; Fig. 1e), refines alignments with a form of L_1_ linear regression (stage IV; Fig. 1e) and finally evaluates the remaining candidates to select the best location to construct a final alignment (stage V). GraphMap computes a BLAST-like *E*-value as well as a mapping quality for its alignments. Further details about each of these stages, the design choices and how they impact GraphMap's performance can be found in the Methods section.

### GraphMap maps reads accurately across error profiles

**Figure 2:**
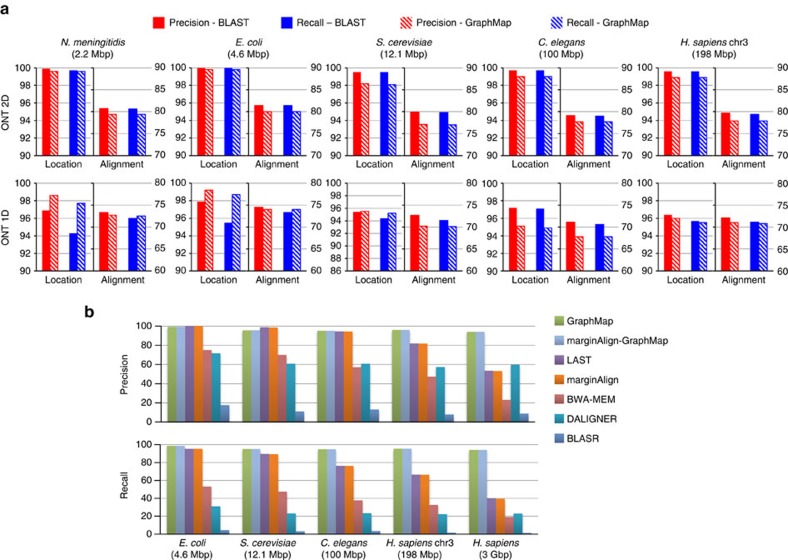
Evaluating GraphMap's precision and recall on synthetic ONT data. (**a**) GraphMap (shaded bars) performance in comparison to BLAST (solid bars) on ONT 2D and 1D reads. Genomes are ordered horizontally by genome size from smallest to largest. For each data set, the graph on the left shows performance for determining the correct mapping location (within 50 bp; *y* axis on the left) and the one on the right shows performance for the correct alignment of bases (*y* axis on the right; Methods section). (**b**) Precision and recall for determining the correct mapping location (within 50 bp) for various mappers on synthetic ONT 1D reads.

GraphMap was designed to be efficient while being largely agnostic of error profiles and rates. To evaluate this feature a wide range of synthetic data sets were generated that capture the diversity of sequencing technologies (Illumina, PacBio, ONT 2D, ONT 1D) and the complexity of different genomes (Fig. 2, Supplementary Fig. 1a). GraphMap's precision and recall was then measured in terms of identifying the correct read location and in reconstructing the correct alignment to the reference (Methods section). These were evaluated separately as, in principle, a mapper can identify the correct location but compute an incorrect alignment of the read to the reference. To provide for a gold-standard to compare against, BLAST (ref. 16) was used as a representative of a highly sensitive but slow aligner which is sequencing technology agnostic. On synthetic Illumina and PacBio data, GraphMap's results were found to be comparable to BLAST (Supplementary Note 1) as well as other mappers (Supplementary Data 1). On synthetic ONT data, we noted slight differences (<3%) between BLAST and GraphMap, but notably, GraphMap improved over BLAST in finding the right mapping location in some cases (for example, for *N. meningitidis* ONT 1D data; Fig. 2a). GraphMap's precision and recall in selecting the correct mapping location were consistently >94%, even with high-error rates in the simulated data. Unlike other mappers, GraphMap's results were obtained without tuning parameters to the specifics of the sequencing technology.

Constructing the correct alignment was more challenging for synthetic ONT data sets and correspondingly the percentage of correctly aligned bases with GraphMap (∼70%) is similar to the number of correct bases in the input data. The use of alternate alignment algorithms and parameters did not alter results significantly (Supplementary Table 1), though the use of a maximum-likelihood based realigner (marginAlign^2^) improved both alignment precision and recall (Supplementary Data 1). The use of marginAlign as a realigner did not improve on GraphMap's ability to identify the correct genomic location (Supplementary Data 1). These results highlight GraphMap's ability to identify precise genomic locations based on robust alignments without the need for customizing and tuning alignment parameters to the unknown error characteristics of the data.

For read-to-reference alignment, programs such as BLAST provide high sensitivity and can be feasible for small genomes, but can quickly become infeasible for larger genomes (for example, runtime for *C. elegans* or the human genome; Supplementary Data 1). Read mappers such as BWA-MEM and BLASR provide a different tradeoff, scaling well to large genomes but with low sensitivity and precision for high-error rates (Fig. 2b, Supplementary Data 1). This could partly be due to specific parameter settings as is the case for BLASR, which was designed for PacBio data. Mappers such as BWA-MEM on the other hand, have different settings optimized for different sequencing technologies (Supplementary Fig. 1b). Despite this, BWA-MEM's performance degrades rapidly even in the ONT setting (Fig. 2b), providing precision and recall <25% for mapping to the human genome (Supplementary Data 1). DALIGNER (ref. 17), a highly sensitive overlapper which additionally supports read mapping, also provided precision and recall that degraded quickly with read error rate and genome size (Fig. 2b, Supplementary Data 1). LAST, originally designed for aligning genomes, fared better in these settings, but still exhibits lower recall for large genomes (30% reduction compared with GraphMap; Fig. 2b) and precision <54% for mapping to the human genome (Supplementary Data 1). The use of a realigner (marginAlign) generally improved alignment precision and recall but results for finding the correct genomic location were similar to that of the original mapper (marginAlign uses LAST by default). GraphMap was the only programme that uniformly provided high sensitivity and recall (Fig. 2b), even for mapping to the human genome, while scaling linearly with genome size (Supplementary Fig. 1c, Supplementary Data 1). Experiments with a range of read lengths and error rates also demonstrate that GraphMap scales well across these dimensions (runtime and memory usage; Supplementary Table 2), though mapping to large genomes currently requires the use of large memory systems (∼100 GB for human genome). Extrapolating this, mapping data from a MinION run of 100,000 reads to the human genome should take <5 h and <$7 on an Amazon EC2 instance (r3.4 × large) using GraphMap.

### Sensitivity and mapping accuracy on nanopore sequencing data

**Figure 3:**
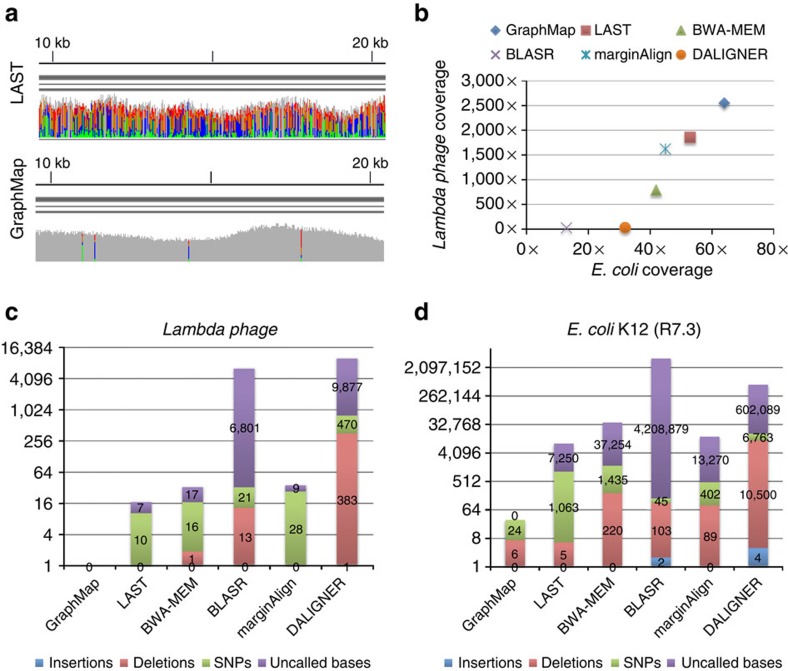
Sensitivity and mapping accuracy on nanopore sequencing data. (**a**) Visualization of GraphMap and LAST alignments for a lambda phage MinION sequencing data set^12^ (using integrative genomics viewer (IGV) (ref. 36)). Grey columns represent confident consensus calls while coloured columns indicate lower quality calls. (**b**) Mapped coverage of the lambda phage^12^ and the *E. coli* K-12 genome^31^ (R7.3 data) using MinION sequencing data and different mappers. (**c**) Consensus calling errors and uncalled bases using a MinION lambda phage data set^12^ and different mappers. (**d**) Consensus calling errors and uncalled bases using a MinION *E. coli* K-12 data set (R7.3) and different mappers.

GraphMap was further benchmarked on several published ONT data sets against mappers and aligners that have previously been used for this task (LAST, BWA-MEM and BLASR; Methods section), as well as a highly sensitive overlapper for which we tuned settings (DALIGNER; Methods section). In the absence of ground truth for these data sets, mappers were compared on the total number of reads mapped (sensitivity), and their ability to provide accurate (to measure precision of mapping and alignment) as well as complete consensus sequences (as a measure of recall). Overall, as seen in the simulated data sets, LAST was the closest in terms of mapping sensitivity compared with GraphMap, though GraphMap showed notable improvements. The differences between GraphMap and LAST were apparent even when comparing their results visually, with LAST alignments having low consensus quality even in a high coverage setting (Fig. 3a). Across data sets, GraphMap mapped the most reads and aligned the most bases, improving sensitivity by 10–80% over LAST and even more compared with other tools (Fig. 3b; Supplementary Fig. 2; Supplementary Note 2). This led to fewer uncalled bases compared with LAST, BWA-MEM, BLASR, DALIGNER and marginAlign even in an otherwise high-coverage data set (Fig. 3c,d). In addition, GraphMap analysis resulted in >10-fold reduction in errors on the lambda phage and *E. coli* genome (Fig. 3c) and reported <40 errors on the *E. coli* genome compared with more than a 1,000 errors for LAST and BWA-MEM (Fig. 3d). With ∼80 × coverage of the *E. coli* genome, GraphMap mapped ∼90% of the reads and called consensus bases for the whole genome with <1 error in 100,000 bases (Q50 quality). The next best aligner, that is, LAST did not have sufficient coverage (20 ×) on >7,000 bases and reported consensus with a quality of ∼Q36. BWA-MEM aligned <60% of the reads and resulted in the calling of >200 deletion errors in the consensus genome. Similar results were replicated in other genomes and data sets as well (Supplementary Fig. 2).

As another assessment of mapping and alignment accuracy, error profiles of 1D and 2D ONT reads were computed for GraphMap and compared with those for LAST and marginAlign. As observed before^2^, substantial variability in the shape and modes of error rate distributions were seen across different mappers, though GraphMap's alignments resulted in lower mismatch rate estimates compared with LAST (Supplementary Fig. 3). GraphMap's distributions were also more similar to those of marginAlign (used as a reference standard), indicating that GraphMap mapping and alignments are at least as accurate as those from LAST. Overall, deletion and mismatch rates for ONT data were observed to be higher than insertion rates, a pattern distinct from the low mismatch rates seen in PacBio data^18^ and explaining why mappers tailored for PacBio data may not work well for ONT data (Supplementary Fig. 3).

Note that the consensus calling results reported here are not comparable to those for programs such as Nanopolish^4^ and PoreSeq^19^, which solve the harder problem of correcting the consensus in the presence of assembly and sequencing errors. To account for a ‘reference bias', where an error-free reference may preferentially enable some programs to report alignments that give an accurate consensus, consensus calling was repeated on a mutated reference (Methods section). Overall, GraphMap was observed to have similar behaviour as other mappers in terms of reference bias, with comparable number of errors (single-nucleotide polymorphisms (SNPs), insertions and deletions) in mutated and non-mutated positions (Supplementary Table 3). These results further confirm that GraphMap's high sensitivity does not come at the expense of mapping or alignment accuracy. In terms of runtime requirements, GraphMap was typically more efficient than BWA-MEM and slower than LAST on these data sets (Supplementary Table 4). Memory requirements were typically <5 GB, with GraphMap and BWA-MEM being intermediate between LAST/BLASR (least usage) and marginAlign/DALIGNER (most usage; Supplementary Data 2).

Analysis of reads that were only mapped by GraphMap when compared with those that were mapped by both GraphMap and LAST revealed characteristics of reads that are more amenable to GraphMap analysis. In particular, these reads were found to be slightly shorter on average (3.4 versus 5.7 kbp), more likely to have windows with higher than average error rate (27 versus 14%), and have a greater proportion of 1D reads (90 versus 76%; *E. coli* R7.3 data set). Overall, GraphMap provided improved sensitivity of mapping on all ONT data sets (Supplementary Note 2), without sacrificing alignment accuracy, and this was further confirmed in the applications discussed below.

### SNV calling in the human genome with high precision

Diploid variant calling using ONT data has multiple potential hurdles including the lack of a dedicated read mapper or diploid variant caller for it^19^. Not surprisingly, a recent report for calling single-nucleotide variants (SNVs) from high-coverage targeted sequencing of the diploid human genome reported that existing variant callers were unable to call any variants and a naive approach requiring 1/3 of the reads to support an allele could lead to many false-positive variants^20^. To evaluate if improved read mappings from GraphMap could increase sensitivity and precision, data reported in Ammar *et al*.^20^ was reanalysed using a rare-variant caller (LoFreq (ref. 3)) that is robust to high-error rates, and compared against a set of gold-standard calls^21^ for this sample (NA12878). Targeted nanopore sequencing reads were mapped by GraphMap to the correct location on the human genome with high specificity, despite the presence of very similar decoy locations (94% identity between *CYP2D6* and *CYP2D7* (ref. 20; Supplementary Fig. 4). GraphMap provided the most on-target reads, aligning 15–20% more reads than the next best mapper (BWA-MEM) for the three amplified genes (*CYP2D6*, *HLA-A* and *HLA-B*; Supplementary Fig. 4). These were then used to call heterozygous variants in these challenging regions of the human genome with high precision (96% with GraphMap; Table 1). GraphMap alignments identified many more true-positive SNVs than other mappers, with comparable or higher precision (76% improvement compared with BWA-MEM and LAST) and a 15% increase in sensitivity over DALIGNER, which has slightly lower precision (93%; Table 1). While the use of a custom variant caller for marginAlign (marginCaller) improved its results in terms of sensitivity, it came at the expense of low precision (36%; Table 1). Subsampling GraphMap mappings to the same coverage as BWA-MEM provided comparable results (42 versus 47 true positives and 2 versus 2 false positives) indicating that GraphMap's improved mapping sensitivity (2 × compared with other mappers) played a role in these results. The ability to sensitively and precisely call SNVs with GraphMap, provides the foundation for reconstructing haplotypes with long reads, and opens up the investigation of complex and clinically important regions of the human genome using nanopore sequencing.

### GraphMap enables sensitive and accurate SV calling

**Figure 4:**
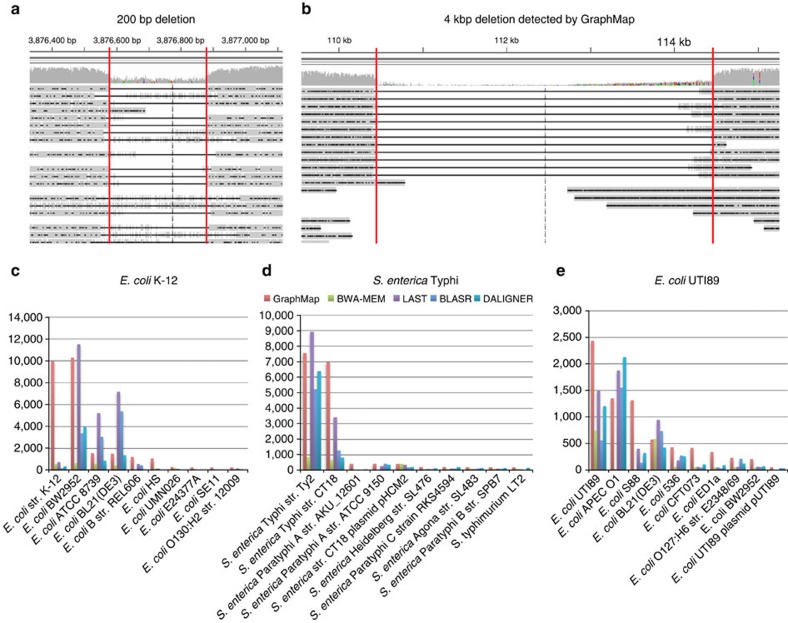
Variant calling and species identification using nanopore sequencing data and GraphMap. (**a**) An IGV view of GraphMap alignments that enabled the direct detection of a 200-bp deletion (delineated by red lines). (**b**) GraphMap alignments spanning a ∼4-kbp deletion (delineated by red lines). Number of reads mapping to various genomes in a database (sorted by GraphMap counts and showing top 10 genomes) using different mappers (GraphMap, BWA-MEM, LAST, DALIGNER and BLASR) and three MinION sequencing data sets for (**c**) *E. coli* K-12 (R7.3) (**d**) *S. enterica* Typhi and (**e**) *E. coli* UTI89. Note that GraphMap typically maps the most reads to the right reference genome (at the strain level) and the *S. enterica* Typhi data set is a mixture of sequencing data for two different strains for which we do not have reference genomes in the database. Results for marginAlign were nearly identical to that of LAST (within 1%) and have therefore been omitted.

Long reads from the MinION sequencer are, in principle, ideal for the identification of large SVs in the genome^22^, but existing mappers have not been systematically evaluated for this application^1^. Read alignments produced by mappers are a critical input for SV callers. To compare the utility of various mappers, their ability to produce spanning alignments or split alignments indicative of a structural variation (insertions or deletions) was evaluated using real *E. coli* data mapped to a mutated reference (Methods section). As shown in Table 2, mappers showed variable performance in their ability to detect SVs through spanning alignments. In comparison, GraphMap's spanning alignments readily detected insertions and deletions over a range of event sizes (100 bp–4 kbp), providing perfect precision and a 35% improvement in recall over the next best mapper (BLASR; Table 2). LAST alignments were unable to detect any events under a range of parameter settings but post-processing with marginAlign improved recall slightly (5%; Table 2). BWA-MEM alignments natively provided 10% recall at 67% precision. Post-processing BWA-MEM alignments with LUMPY improved recall to 45%, using information from split reads to predict events. GraphMap produced spanning alignments natively that accurately demarcated the alignment event and did this without reporting any false positives (Fig. 4a,b and Table 2).

### Sensitive and specific pathogen identification with ONT data

Due to its form factor and real-time nature, an application of MinION sequencing that has garnered interest in the community is in the identification of pathogens in clinical samples. Sequencing errors (particularly in 1D data) and the choice of read mapper could significantly influence results in such an application and lead to misdiagnosis. GraphMap's high specificity in read mapping as seen in the results for Ammar *et al*. (Supplementary Fig. 4) suggested that it could be useful in this setting. Clonal sequencing data on the MinION and a database of microbial genomes was used to create several synthetic benchmarks to evaluate the performance of various mappers for this application (Methods section). For species level identification, all mappers reported high precision (typically >95%) but recall varied over a wide range from 20 to 90% (Table 3). GraphMap had the highest recall and F_1_ score in all data sets, providing an improvement of 2–18% over other mappers. The improvement was more marked when a perfect reference was not part of the database (for example, *S. enterica* Typhi, Table 3) For this application, BWA-MEM was the next best mapper while LAST and BLASR exhibited >25% reduced recall compared with GraphMap (Table 3). Not surprisingly, strain-level identification using MinION data appears to be much more difficult and in some cases a closely related strain can attract more reads than the correct strain (Fig. 4c). However, in the data sets tested, GraphMap assigned most reads to a handful of strains that were very similar to the correct strain (Fig. 4c–e; 99.99% identity for *E. coli* K-12 and BW2952). Moreover, the use of strain-specific sequences was able to unambiguously identify the correct strain from this subset (for example, there were no reads mapping to NC_012759.1:4.13–4.17 Mbp, a region unique to BW2952), indicating that this approach could be used to systematically identify pathogens at the strain level.

## Discussion

The design choices in GraphMap, including the use of new algorithmic ideas such as gapped spaced seeds, graph mapping and longest common subsequence in k Length substrings (LCAk), provide a new tradeoff between mapping speed and sensitivity that is well-suited to long nanopore reads. For mapping error-prone synthetic long reads to the human genome, GraphMap was the only mapper that exhibited BLAST-like sensitivity, while being orders of magnitude faster than BLAST. On nanopore sequencing data from the MinION system, GraphMap was unmatched in terms of sensitivity, mapping >90% of reads and 95% of bases on average. Compared with other mappers, this lead to a 10–80% increase in mapped bases (for example, 18% increase on a recent MinION MkI data set; Supplementary Note 2). This is a significant improvement—typically mapping programs are highly optimized and increase in sensitivity of even a few percentage points can be hard to achieve. Additionally, sensitivity is a key requirement for mapping tools and mapping-based analysis, as reads that cannot be mapped are unavailable for use in downstream applications. A drawback of the current implementation of GraphMap is the requirement of large-memory machines for mapping to large genomes (∼100 GB for the human genome). The use of more memory-efficient index structures (for example, FM-index) can significantly reduce this requirement (for a modest increase in runtime) and this option is currently under implementation.

GraphMap's speed and sensitivity do not come at the expense of location and alignment precision, as demonstrated by extensive experiments with synthetic and real data sets. For determining the correct genomic location, GraphMap's precision is typically >98% and it is able to distinguish between candidate locations that are >94% identical on the human genome. For alignment precision, GraphMap's performance scales according to sequencing error rate, is comparable to BLAST and other mappers (BWA-MEM, LAST, BLASR and DALIGNER), and was observed to be robust to the choice of alignment algorithms and parameters. GraphMap mappings provided a better starting point for the realigner marginAlign^2^ and should do so for consensus calling algorithms such as Nanopolish^4^ and PoreSeq^19^ as well.

In general, GraphMap's improved sensitivity should benefit a range of applications for nanopore data and a few of these were explored in this study. In particular, variant calling and species identification with error-prone data can be affected by errors in mapping and alignment. Despite the lack of custom variant callers, read mappings from GraphMap were shown to provide sensitive and precise SNV calls on complex regions of the human genome. In addition, GraphMap alignments readily spanned insertions and deletions over a wide range of sizes (100 bp–4 kbp) allowing for the direct detection of such events, without assembly or split read analysis. With the development of new nanopore-specific variant calling tools, GraphMap's improved sensitivity should continue to provide a useful starting point for these applications. Furthermore, GraphMap alignments were used to identify the species-level origin of reads with high precision and recall. The sensitivity of mapping with GraphMap can be a key advantage in applications where MinION sequencing reads are used in real-time to identify pathogens^23^, particularly in combination with rapid protocols for generating 1D reads on the MinION. With further downstream processing, these read mappings could be used for strain-level typing and characterization of antibiotic resistance profiles^23^, meeting a critical clinical need.

In principle, the approach used in GraphMap could be adapted for the problem of computing overlaps and alignments between reads. As was recently shown, nanopore sequencing reads can be used to construct high-quality assemblies *de novo*^4^ and sensitive hashing techniques have been used for the assembly of large genomes^24^. GraphMap's sensitivity and specificity as a mapper could thus serve as the basis for fast computation of overlap alignments and *de novo* assemblies in the future.

## Methods

### Description of the GraphMap algorithm

*Region selection*. GraphMap starts by roughly determining regions on the reference genome where a read could be aligned. This step is performed to reduce the search space for the next step of the algorithm, while still providing high sensitivity. As a first step, region selection relies on finding seeds between the query sequence and the reference, before clustering them into candidate regions. For seed finding, commonly used approaches such as maximal exact matches (MEMs; as used in BWA-MEM (ref. 10)) or Hamming distance based spaced seeds^24^,^25^ (as used in LAST (ref. 14)) were found to be either not sensitive enough or not specific enough in the presence of error rates as high as is feasible in nanopore data (for example, see ‘Fixed seed *k*=13' for ONT 1D data in Supplementary Data 3). Instead, a form of gapped spaced seeds was employed, similar to gapped q-gram filters for Levenshtein distance^15^. Specifically, the approach proposed in Burkhardt and Kärkkäinen^15^ was extended to use both one- and two-gapped q-grams (Fig. 1b) as detailed below.

Gapped q-grams are a seeding strategy that allow for fast and very sensitive lookup of inexact matches, with variations allowed in predefined ‘do not care' (DC) positions of the seed. Consistent with existing terminology, the concrete layout of the inclusive and DC bases is referred to here as a shape and the number of used positions its weight. Gapped q-grams allow for DC positions within a shape to also contain insertions and deletions (indels). The approach in GraphMap for implementing Levenshtein gapped q-grams is based on constructing a hash index of the reference sequence, where the q-gram positions are hashed by the keys constructed from the shape's layout—only inclusive bases are taken for constructing the key, while the DC bases are simply skipped (Fig. 1b). During the lookup step, multiple keys are constructed for each shape and used for retrieval. For each DC base, three look-up keys are constructed:

1. A key constructed in the same manner as during the indexing process, which captures all seed positions and with a DC base being a match or a mismatch (for example, ‘1110111'; see ‘(Mis)match seed' in Fig. 1b),
2. A key where the DC base is not skipped. This key captures up to one deletion (as indels are frequently 1 bp long) at the specified position (for example, ‘111111'; see ‘Deletion seed' in Fig. 1b), and
3. A key where the DC base as well as the following base is skipped. This key allows for at most one insertion and one match/mismatch (for example, ‘11100111'; see ‘Insertion seed' in Fig. 1b).

In total, for each shape *d*^3^ keys are constructed, where *d* is the number of DC bases. GraphMap uses two complementary shapes for the region selection process: ‘1111110111111' (or the 6-1-6 shape) and ‘11110111101111' (or the 4-1-4-1-4 shape), where 1 marks the inclusive bases and 0 the DC positions. This shape combination was selected based on empirical evaluation of a range of combinations, due to the computational intractability of computing the optimal shape for the Levenshtein distance^25^,^26^ (see Supplementary Data 3 for results for each shape and the combination). For each shape, a separate index is used in GraphMap. At every seed position, both shapes are looked up, and all hits are used in the next step for binning.

To derive a general approach for binning seed hits, we draw on the concept of a Hough transform (HT), a method commonly used in image processing for detection of shapes such as lines, circles and ellipses. The HT defines a mapping from image points into an accumulator space, called the Hough space. In the case of line detection, if a given set of points in Cartesian space are collinear, then their relation can be expressed with a linear equation with common slope *m* and intercept *c*:


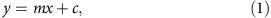


where (*x*, *y*) are the coordinates of a point in 2D space. HT attempts to determine parameters *m* and *c* of a line that describes the given set of points. Note that the system is generally over-determined and thus the problem can be solved using linear regression techniques. However, the HT uses an evidence-gathering approach, which can be used to detect an arbitrary number of lines in the image instead of only one best (Fig. 1c). Equation (1) can be converted into its dual in parameter space:


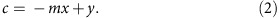


The intuition is as follows: given a point (*x*, *y*) in Cartesian space, its parameter space representation defines a line. If multiple Cartesian space points are given, each transforms into a different line in the parameter space. Their intersections specify potential lines in the original, Cartesian space. HT defines an accumulator space, in which *m* and *c* are rasterized so as to take only a finite range of values. HT then simply counts all the potential solutions in the accumulator space by tracing all the dual lines for each point in the Cartesian space, and increasing the vote count for each (*m*, *c*) coordinate. All HT space coordinates with count above a defined threshold can then be considered as candidate lines in the original Cartesian space.

A single-seed hit can be represented with a ‘*k*-point' (*q*, *t*) in 2D space, where *q* is the seed's position on the read, and *t* is the position of the seed hit on the reference. In the case a read is completely error-free and extracted from the exact reference, its set of *k*-points would be perfectly collinear in such defined space. Moreover, under these ideal conditions, they would all lie on a line tilted at a 45° angle (slope *m*=1). This collinearity also corresponds to the main diagonal in the dynamic programming alignment matrix. Since *m* is known, only the intercept parameter *c* needs to be determined to find the accurate mapping position. As *c* corresponds to the (already discrete) coordinates on the reference sequence, a simple integer array of the length of the reference can be used for counting votes (Fig. 1c). For each k-point, its *c* parameter value is determined with a simple expression:


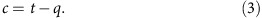


The index of the accumulator array with the highest count is the exact mapping position of the read on the reference. In this simple form, this approach mirrors the techniques used in other aligners (for example, FASTA). However, the concept of the HT allows us to extend and generalize this notion.

We account for substitution and indel errors in this framework as follows: substitution errors cause only the reduction in the maximum vote count for the correct *c* value and induce noise votes in other locations on the reference. Such type of errors can be addressed using appropriate thresholding on the hit count (see below). On the other hand, indels are of special interest because they shift the alignment diagonal and cause more substantial reduction of votes for the correct location. Additionally, using an accumulator array that is of size equal to the size of the reference sequence can cause high memory consumption, especially in the case of processing large sequences in multithreaded environments.

To address both the error-rate and memory consumption issues, GraphMap rasterizes the reference sequence into partitions of length *L*/3 (where *L* is the read length), so that at least one partition is fully covered by the read. For each seed hit to a bin, it increments the value of the bin corresponding to its *c* parameter value determined using equation (3). Bins are then sorted in descending order of the number of hits. To limit the search to the most likely bins, only bins with count >75% of the max count are selected for further processing. A region is then defined as a portion of the reference that expands the corresponding bin's start and end location by an additional read length, to compensate for potential indel errors and ensure that the entire alignment area enters the next step of mapping. In the case that the reference genome is specified as being circular by the user, GraphMap allows the region to be constructed by concatenating the beginning and the end of the reference. Regions are then processed separately until the last step of the method, when the highest scoring region is selected for alignment.

*Graph-based vertex-centric construction of anchors*. In this stage, candidate regions from stage I are refined by constructing alignment chains or anchors from short seeds matches. To do this, GraphMap uses the notion of a ‘kmer mapping graph'. Given a pair of sequences (target and query), it starts by constructing a kmer mapping graph from the target sequence. In the current implementation, the read was chosen to be the target sequence to reduce memory consumption. The vertices of the kmer mapping graph are the kmers of the target sequence of length *T* (Fig. 1d). Unlike in a de Bruijn graph, identical kmers are not truncated into the same vertex of the graph but are kept as separate individual vertices (Fig. 1d). For every vertex 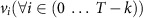, *l* directed outbound edges are added which connect *v*_*i*_ to vertices *v*_*i*+1_,*v*_*i*+2_,…,*v*_*i*+*l*_ (Fig. 1d). The rationale for such a design is as follows: in case *l*=1 and if the query is a subset of the target with no differences or errors, the target's mapping graph would contain the same kmers in the exact same order as in the query sequence. Thus, an exact walk exists in both sequences. However, in realistic conditions, variations and sequencing errors exist in reads. Although the majority of kmers might still be in the same order, a simple exact linear walk through the reference's and read's mapping graphs cannot be found due to the differing kmers present. Instead, the walk is fragmented into several smaller ones and this is particularly severe when the error rate is high, as seen in nanopore sequencing. To address this issue, the additional (*l*−1) edges act as a bridge between vertices in the mapping graph. Thus GraphMap allows a linear walk to be found not only by following consecutive kmers in the graph, but to jump-over those that produce poorer solutions. Figure 1d depicts such an example. GraphMap uses *l*=9 by default as it was empirically found to enable anchor construction for most ONT reads.

For graph construction, GraphMap uses an index constructed from the target on the fly, using a smaller continuous seed for sensitivity (default *k*=6, similar to the kmer used for MinION base-calling). In principle, any indexing method can be used and for runtime efficiency GraphMap uses perfect kmer hashing when *k*<10 and suffix arrays otherwise. To do graph traversal, for each consecutive kmer in the query, a list of hits on the target sequence is obtained from the index. The vertex-centric walk then works as follows: for a chosen vertex, collect information from input edges, choose the ‘best' edge and update the information it contains, and transmit this information to all outbound edges simultaneously. The ‘best' edge is defined here to be the one belonging to the longest walk. The information that is transmitted through the edges contains the walk length, the position of the starting kmer in both the target and the read, and the number of covered bases and kmers in both sequences. Thus the runtime complexity of the vertex-update operation is O(1).

After all kmers from the query have been processed, a list of walks in the graph is collected. Walks that are too short (default<12 bases, that is, smaller than the seeds from stage I) are excluded to avoid a large search space. Vertex-centric walks allow GraphMap to quickly construct longer alignments in the presence of higher substitution error rates, as seen in nanopore sequencing data. In the presence of low-substitution error rates (<2%, as is the case for Illumina as well as PacBio reads), a single walk can cover most of, if not the entire read. For ONT reads we observed shorter walks that we refer to here as anchors (Fig. 1d).

*Extending anchors into alignments using LCSk*. Each anchor reported by GraphMap in stage II represents a shared segment (or subsequence) between the target and the query sequence with known start and end positions in both sequences. Due to the presence of repeats, the set of anchors obtained is not necessarily monotonically increasing in both the target and query coordinates. For this reason, a subset of anchors that satisfy the monotonicity condition needs to be selected. The problem of identifying such a subset can be expressed as finding the Longest Common Subsequence in k Length Substrings^27^ (LCSk). Note that this is distinct from just finding the longest common subsequence as that ignores the information determined in the anchors and can favour alignments that have many more indels. Recently, an efficient and simple algorithm for solving a variant of the LCSk problem has been proposed^28^. In our implementation we follow this paradigm and instead of using substrings of fixed size *k*, we allow for variable length substrings. Concretely, the size of each substring is equal to the length of the corresponding anchor in both sequences. As a result, the reconstruction of LCSk is obtained in the form of a list of consecutive anchors in the target and the query sequence. The LCSk stage was observed to be key to GraphMap's ability to construct approximate alignments that help identify the correct mapping location. Removing this stage reduced GraphMap's precision and recall by 10–30% without significantly affecting its runtime or memory usage (Supplementary Data 3).

*Refining alignments using L*_*1*_ *linear regression*. The alignments obtained using LCSk tend to be largely accurate but since its definition lacks constraints on the distance between substrings, the alignments obtained may include outlier matches and incorrect estimation of overall alignment length (Fig. 1e). These outliers are caused by repeats or sequencing errors, but they still satisfy the monotony condition. Similar to the observation presented for region selection, the LCSk list of anchors should ideally be collinear in the 2D query-target coordinate space, with a slope of 45°. All deviations from this line are caused by indel errors, and can be viewed as noise. The filtering of outlier anchors begins by fitting a 2D line with a 45° slope in the query-target space under the least absolute deviation criteria (L_1_). Next, a subset of anchors which are located within 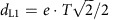 from either side of the L_1_ line is selected, where *e* is the expected error rate (by default, conservatively set to 45%), *T* is the target (read) length and the factor 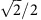 is used to convert the distance from target coordinate space to a distance perpendicular to the L_1_ line. A confidence interval 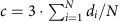 is calculated, where *d*_*i*_ is the distance from a selected anchor *i* to the L_1_ line (the constant 3 was chosen to mimic a 3σ rule). LCSk is then repeated once again but only on the anchors which are located within the distance ±*c* from the L_1_ line to compensate for possible gaps caused by anchor filtering (Fig. 1e). The use of L_1_ filtering was observed to improve the precision of alignment start and end coordinates for many reads, though the overall impact on performance was less significant in comparison to the LCSk stage (Supplementary Data 3).

After filtering, five empirically derived scores that describe the quality of the region are calculated. They include: the number of exact kmers covered by the anchors *n*_kmers_, the s.d. *σ* of anchors around the L_1_ line, the length of the query sequence which matched the target (distance from the first to the last anchor) *m*_len_, the number of bases covered by anchors (includes only exact matching bases) *n*_cb_ and the read length. The last four scores are normalized to the range [0,1] with the following equations (4, 5, 6, 7):


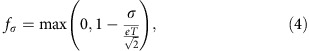



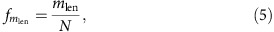



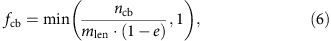



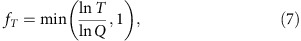


where *Q* is the length of the reference sequence (query in our previous definition). The overall quality of the alignment in a region is then calculated as the product of the normalized scores:


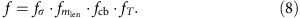


*Construction of final alignment*. After all selected regions have been processed they are sorted by the *f* value. The region with the highest value *f*_max_ is selected for the final alignment. The default settings for GraphMap use an implementation of Myers' bit-vector algorithm for fast alignment^29^. GraphMap also allows users a choice of aligners, including an implementation of Gotoh's semi-global alignment algorithm^30^, as well as an option to construct anchored alignments. Specifically, in the anchored approach, anchors from the LCSk step are clustered and alignments within and between cluster end points computed using Myers' bit-vector alignment (extensions to read ends are done without gap penalty). Clustering is done by collecting neighbouring anchors where the ratio of distances in the read and reference coordinates is <*e*/2 (as before, *e* is the expected error rate in the data, and the factor of 2 allows for more stringent clustering). Clusters with very few bases (<30 or 2% of read length) were discarded for this purpose as they were found to reduce alignment accuracy.

GraphMap allows users to output all equally or similarly good secondary alignments by specifying an ambiguity factor *F* in the range [0,1] and using that to select regions which have *n*_kmers_≥(1−*F*)·*n*_kmers,best_, where *n*_kmers,best_ is the number of kmers of the region with the maximum *f* value. We denote the count of regions with *n*_kmers_ above the ambiguity threshold as *N*_a_.

*Mapping quality*. Since the region filtering process in GraphMap maintains a large collection of possible mapping positions on the given reference, it enables meaningful calculation of the mapping quality directly from its definition:


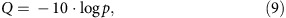


where *p* is the probability of the read being mapped to the wrong position. We calculate *p* simply as 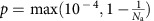, that is, max *Q*=40, and report quality values according to the sequence alignment/map (SAM) format specification.

*E-value*. For each reported alignment, GraphMap calculates the *E*-value which is given as a custom ‘ZE' parameter in the output SAM file. Following the approach used in BLAST, we rescore alignments and use pre-calculated Gumbel parameters to compute *E*-values in the same way as in BLAST (default parameters from BLAST: match=5, mismatch=−4, gap_open_=−8 and gap_extend_=−6).

### Data sets

For evaluating GraphMap and other tools, we used eight publicly available MinION sequencing data sets, 49 synthetic data sets and MinION sequencing reads for an *E. coli* UTI89 sample as detailed below.

*MinION library preparation*. Genomic DNA was extracted from *Escherichia coli* UTI89 using the QIAamp DNA mini kit (Qiagen). Extracted DNA (1 μg) was then sheared in a total volume of 80 μl using a Covaris g-TUBE according to the manufacturer's instructions with centrifugation for 1 min at 6,000 r.p.m. Sheared DNA was end repaired and A-tailed using the GeneRead DNA Library Prep I Kit from Qiagen according to the manufacturer's protocol. The reaction was purified using 1 × volume of Agencourt Ampure XP beads and eluted in 30 μl nuclease-free water. Subsequent steps of DNA sequencing library preparation were carried out using Oxford Nanopore's MinION Genomic DNA Sequencing Kit (SQK-MAP003) according to the manufacturer's recommended protocol, including the addition of purified BSA (NEB) to Agencourt Ampure XP beads and Elution buffer.

*MinION sequencing of E. coli UTI89*. Immediately before sequencing, 12 μl of the DNA library was combined with 134 μl EP buffer and 4 μl Fuel Mix and mixed by inversion 10 times. The flow cell was primed by washing with two aliquots of 150 μl of EP buffer, with 10 min in between washes. Prepared DNA Library (150 μl) was then loaded onto the flow cell and the Genomic DNA 48 h sequencing run programme was selected. Fresh sample was loaded onto the flow cell at 12 h intervals throughout the run.

*Publicly available sequencing data sets*. Eight publicly available MinION sequencing data sets were used for evaluation. These include a lambda phage data set, three *E. coli* data sets (each produced with a different version of MinION chemistry), reads for *S. enterica* Typhi, *A. bayalyi* ADP1 and *B. fragilis* BE1, and a data set consisting of three amplicons from the human genome, as detailed below:

1. Lambda phage burn-in data set^12^. The data set consists of 40,552 reads in total (211 Mbp of data), generated using an early R6 chemistry. The reference genome (NC_001416) is 49-kbp-long giving an expected coverage of >4,300 × .
2. *E. coli* K-12 MG1655 R7 data set^31^. The data set has 111,128 reads (668 Mbp) providing 144 × coverage of a 4.6-Mbp genome (U00096.2).
3. *E. coli* K-12 MG1655 R7.3 data set^31^. The data set has 70,531 reads (311 Mbp) providing 67 × coverage of the genome (U00096.2).
4. *E. coli* K-12 MG1655 SQK-MAP006-1 data set. The data set consists of 116,635 reads (1.06 Gbp) providing 228 × coverage of the genome (U00096.2). Sequencing was performed in four runs: two with natural DNA, and two with a low-input library that includes a PCR step. The data set used in this paper consists of the first natural DNA run (MAP006-1; http://lab.loman.net/2015/09/24/first-sqk-map-006-experiment/).
5. *S. enterica* Typhi data set^1^. The data set is composed of two runs of strain H125160566 (16,401 and 6,178 reads, respectively) and one run of strain 08-04776 (10,235 reads). When combined, this data set consists of 32,814 reads (169 Mbp) which amounts to 35 × coverage of a closely related reference sequence, *S. enterica* Typhi Ty2 (NC_004631.1; 4.8 Mbp genome).
6. *A. baylyi* ADP1 data set^8^. The data set consists of 66,492 reads (205 Mbp) providing 57 × coverage of a 3.6-Mbp genome (NC_005966.1).
7. *B. fragilis* BE1 data set^7^. The data set consists of 21,900 reads (141 Mbp) providing 27 × coverage of a 5.2-Mbp genome (LN877293.1 assembly scaffold).
8. Amplicon sequencing of human *HLA-A*, *HLA-B* and *CYP2D6* genes^20^. The data set contains 36,779 reads in total. As a reference, chromosomes 6 and 22 from hg19 GRCh37 *H. sapiens* reference were used^20^.

*Synthetic data sets*. Synthetic Illumina reads were generated using the ART simulator^32^ (150 bp single-end) and PacBio continuous long reads (CLR) reads using the PBSIM simulator^18^ (with default settings). For synthetic MinION data we adopted PBSIM (as no custom ONT simulators exist currently) and used parameters learnt from LAST alignments (to avoid bias towards GraphMap) with *E. coli* K-12 R7.3 data (Supplementary Table 5). Reads were simulated (*n*=1,000) for six reference sequences: *N. meningitidis* serogroup A strain Z2491 (1 chromosome, 2.2 Mbp, NC_003116.1), *E. coli* K-12 MG1655 (1 chromosome, 4.6 Mbp, U00096.2), *S. cerevisiae* S288C (16 chromosomes, 12 Mbp), *C. elegans* (6 chromosomes, 100 Mbp), *H. sapiens* Chr3 (198 Mbp, GRCh38, CM000665.2) and the entire *H. sapiens* genome (3.1 Gbp, GRCh38).

To estimate GraphMap's scalability with respect to error rate and read length, 25 additional data sets were simulated from the *S. cerevisiae* S288C reference, q has been added each pair (*e*, *L*) of error rate *e*∈{5, 10, 15, 20 and 25}% and read lengths *L*∈{1, 2, 3, 4 and 5} kbp (*n*=10,000).

### Evaluation methods

*Performance on synthetic data*. Mappers were evaluated for precision and recall in meeting two goals:

1. Finding the correct mapping location—a read was considered correctly mapped if its mapping position was within ±50 bp of the correct location. In case an alignment contained soft- or hard-clipped bases, the number of clipped bases was subtracted from the reported alignment position to compensate for the shift.
2. Reporting the correct alignment at a per-base-pair level—a base was considered correctly aligned if it was placed in exactly the same position as it was simulated from. Unaligned reads and soft- or hard-clipped portions of aligned reads were not taken into account for precision calculation. Recall was calculated with respect to all simulated bases in reads.

*Parameter settings for mappers*. BWA-MEM was evaluated with the nanopore setting (- × ont2d) unless otherwise stated (version:bwa-0.7.12-r1034, commit: 1e29bcc). BLASR was evaluated with the options ‘-sam -bestn 1' (version: 1.3.1, commit: f7bf1e5) and in addition for the database search we set more stringent parameters (‘-minMatch 7 -nCandidates 1'). LAST was run with a commonly used nanopore setting^31^ (‘-q 1 -r 1 -a 1 -b 1'; version: 475). BLAST (version: ncbi-blast-2.2.30+- × 64-linux) was run with default settings for Illumina data and a more suitable nanopore setting^33^ ‘-reward 5 -penalty -4 -gapopen 8 -gapextend 6 -dust no' for ONT and PacBio data. GraphMap (version: v0.21, commit: 0bd0503) was run with default settings. In addition, for circular genomes we used the -C option, anchored alignments for calling structural variations (‘-a anchor') and *E*-value filtering (‘-z 1e0') for database search and variant calling. marginAlign was run with the ‘—em' option on each data set to estimate the correct parameters since data quality varied across data sets (commit: 10a7a41). In the case of simulations, the model parameters were first calculated for every simulated data type using a sample data set, and then marginAlign was run using corresponding models. Furthermore, since marginAlign is a realigner and uses a mapper for seeding the alignment position, we forked and expanded marginAlign to create a version that uses GraphMap instead of LAST as its seed mapper. Our modified version of marginAlign is available on GitHub: https://github.com/isovic/marginAlign (commit: d69264d). The modified version of marginAlign was also used with the ‘—em' option, with the additional parameter ‘—graphmap' to use GraphMap. We also compared against DALIGNER (commit: d4aa487). For synthetic data, DALIGNER was tested using three combinations of parameters: default, ‘-e.7 -k10' and ‘-e.7 -k9'. As ‘-e.7 -k10' was found to have the best results for synthetic ONT data (Supplementary Data 1), it was used for all tests on real nanopore data.

*Consensus calling using MinION data*. Consensus was called using a simple majority vote of aligned bases, insertion and deletion events (insertion sequences were taken into account while counting events) and positions with <20 × coverage were not called. Our consensus caller is implemented in a script ‘consensus.py' that is freely available at https://github.com/isovic/samscripts. All reads were mapped to just the corresponding reference and analysed to determine consensus sequences. The *E. coli* K-12 reference was mutated using Mutatrix (https://github.com/ekg/mutatrix) with parameters ‘—snp-rate 0.0006—population-size 1—microsat-min-len 0—mnp-ratio 0—indel-rate 0.0067—indel-max 10' to emulate the draft nanopore-only assembly reported by Loman *et al*.^4^ (∼3,750 SNPs and ∼42,500 indels). Real nanopore reads were mapped to the mutated reference, and consensus variants (from ‘consensus.py') were used to construct a consensus sequence with GATK's FastaAlternateReferenceMaker tool (GATK version 3.4–46). Consensus sequences were compared with the original reference using nucmer and dnadiff^34^ (MUMmer 3.0). Positions±2 bp from the mutated position were also considered in calculating consensus errors in mutated positions to account for alignment uncertainty in homopolymer sequences.

*Benchmarking mappers for pathogen identification*. Bacterial genomes related to a list of water-borne pathogens were selected from NCBI's bacterial database to construct a database of 259 genomes (550 Mbp; Supplementary Data 4). MinION sequencing data sets from cultured isolates were used as proxy for sequencing of pathogen-enriched clinical samples (using data for *E. coli* K-12 R7.3, *S. enterica* Typhi and *E. coli* UTI89, as specified earlier). This is a simple test case as real samples are likely to have contamination from other sources as well (for example, human DNA). We mapped these three read data sets to the database of bacterial genomes using each of the mappers to find unique alignments and test if these could help identify the correct species and strain. For BWA-MEM, LAST, marginAlign and DALIGNER, we chose the best alignment based on alignment score (AS; as long as AS and mapping quality were >0) and for GraphMap and BLASR we used the unique reported alignment (mapping quality>0). Since marginAlign and DALIGNER do not report the AS in their output, we rescored their alignments (parameters match=1, mismatch=−1, gap_open_=−1 and gap_extend_=−1) to make them comparable.

*Single-nucleotide variant calling*. All 2D reads from Ammar *et al*.^20^ were mapped to the human genome (GRCh37.p13; chr 6 and 22) and for each read only the alignment with the highest AS was kept. To avoid chimeric reads as reported in the original study only reads that fully spanned the amplicon regions were used for this analysis. Variants were called using LoFreq (ref. 3; version: 2.1.2) with the parameters ‘-a 0.01 -q 0 -Q 0—no-default-filter'. A custom caller for marginAlign (marginCaller) was also used to call SNVs. The detected SNVs were then compared with known variants from dbSNP and a high-confidence set for NA12878 (ref. 21; the sequenced sample; ftp://ftp.ncbi.nlm.nih.gov/snp/organisms/human_9606_b141_GRCh37p13/VCF/All.vcf.gz; ftp-trace.ncbi.nih.gov/giab/ftp/data/NA12878/variant_calls/NIST/NISTIntegratedCalls_14datasets_131103_allcall_UGHapMerge_HetHomVarPASS_VQSRv2.18_all.primitives.vcf.gz) to identify true positives and false positives.

*Structural variation detection*. We modified the *E. coli* K-12 MG1655 reference by inducing 20 SV events (10 insertions and 10 deletions) of different sizes: 100, 300, 500, 1,000, 1,500, 2,000, 2,500, 3,000, 3,500 and 4,000 bp. All 2D reads from both *E. coli* K-12 data sets (R7 and R7.3) were combined and mapped. SVs were detected by simple consensus vote of indel events reported in spanning alignments (≥20 bases to avoid sequencing errors). In the absence of a sophisticated SV caller for nanopore data we used a simple rule that identifies windows where >15% of the reads at each position report an insertion (or deletion) event (at least five reads). To avoid fragmented events due to a local drop in allele frequency, windows which were less than window-length apart (max of the two windows) were merged. A detected event was considered a true positive if its size was within a 25% margin of the true size and its start and end locations were <25% of event size away from the true locations. LUMPY (ref. 35; version: 0.2.11) was used for testing the use of split read alignments. The script ‘extractSplitReads_BwaMem' provided with LUMPY was used to extract split reads from BWA-MEM alignments. As the default setting (‘minNonOverlap=20') did not report any results, the script was run with the setting ‘minNonOverlap=0' to allow split alignments to be adjacent on the read.

### Code availability

GraphMap is available open source under the MIT license at https://github.com/isovic/graphmap. Scripts to reproduce all results in this study can be found at https://github.com/isovic/graphmap-reproduce.

## Additional information

**Accession codes:** The MinION sequencing of E. coli UTI89 was deposited in the European Nucleotide Archive under the accession code ERX987748.

**How to cite this article:** Sović, I. *et al*. Fast and sensitive mapping of nanopore sequencing reads with GraphMap. *Nat. Commun.* 7:11307 doi: 10.1038/ncomms11307 (2016).

## Supplementary Material

Supplementary Information Supplementary Figures 1-4, Supplementary Tables 1-5, Supplementary Notes 1-2 and Supplementary References

Supplementary Data 1 Summarized performance data for different mappers across a range of simulated datasets

Supplementary Data 2 Summarized performance data for different mappers across a range of real nanopore datasets

Supplementary Data 3 Summarized performance data for different design choices in GraphMap across a range of simulated datasets

Supplementary Data 4 List of bacterial genomes used for a pathogen identification test

## Figures and Tables

**Table 1:** Comparison of various mappers for SNV calling.

	LAST	marginAlign	BWA-MEM	BLASR	DALIGNER	GraphMap
Precision (%)	94	100 (36)	96	100	93	96
True positives	49	1 (107)	47	43	75	86

Results are based on amplicon sequencing data for a human cell line (NA12878) for the genes *CYP2D6*, *HLA-A* and *HLA-B*. Precision values are likely to be an underestimate of what can be expected genome-wide due to the repetitive nature of the regions studied and the incompleteness of the gold-standard set. Results for marginAlign using marginCaller are shown in parentheses.

**Table 2:** Comparison of various mappers for structural variant calling.

	LAST	marginAlign	BWA-MEM	BLASR	DALIGNER	GraphMap
Precision (%)	0	50	67 (90)	94	0	**100**
Recall (%)	0	5	10 (45)	75	0	**100**
F_1_ score (%)	0	9	17 (60)	83	0	**100**

Results are based on mapping a MinION data set for *E. coli* K-12 (R7.3) on a mutated reference containing insertion and deletions in a range of sizes ((100 bp, 300 bp, 500 bp, 1 kbp, 1.5 kbp, 2 kbp, 2.5 kbp, 3 kbp, 3.5 kbp and 4 kbp); 20 events in total). Bold values indicate the best results for each metric. The F_1_ score is given by a weighted average of precision and recall. Values in parentheses for BWA-MEM show the results using LUMPY.

**Table 3:** Precision and recall for species identification using MinION reads.

	*E. coli* K-12 (R7.3)			*S. enterica* Typhi			*E. coli* UTI89		
	Prec.	Rec.	F_1_	Prec.	Rec.	F_1_	Prec.	Rec.	F_1_
BLASR	93	22	36	**99**	28	44	98	55	70
LAST	94	37	53	97	34	51	95	65	78
DALIGNER	80	10	17	**99**	28	43	98	55	71
BWA-MEM	94	47	63	98	45	61	98	85	91
GraphMap	**95**	**51**	**67**	97	**56**	**72**	**99**	**88**	**93**

Bold values indicate the best results for each data set and metric. Results for marginAlign were nearly identical to that of LAST (within 1%) and have therefore been omitted.
