# Supplementary material for: Fast and sensitive mapping of nanopore sequencing reads with GraphMap
Source: Nat Commun. 2016 Apr 15;7:11307. doi: 10.1038/ncomms11307 (PMC4835549; doi:10.1038/ncomms11307)
Supplement: Supplementary Information — Supplementary Figures 1-4, Supplementary Tables 1-5, Supplementary Notes 1-2 and Supplementary References [file ncomms11307-s1.pdf]

Supplementary Figure 1. Performance evaluation on synthetic datasets

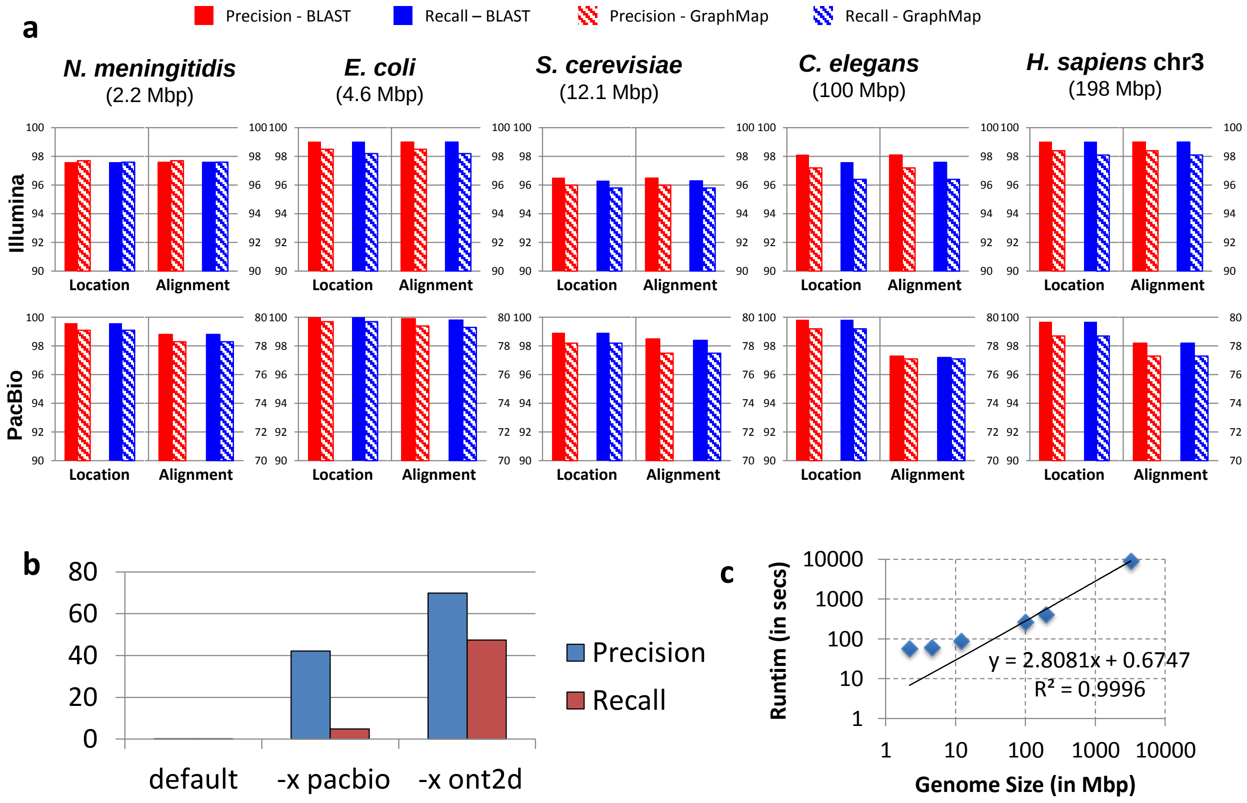

(a) GraphMap compared to BLAST on synthetic Illumina and PacBio reads (see **Fig. 2a**) (b) BWA-MEM location results with different settings (*S. cerevisiae* genome; 1D reads) (c) Runtime scalability for GraphMap (1D reads).

Supplementary Figure 2. Consensus calling errors and uncalled bases using MinION datasets and different mappers

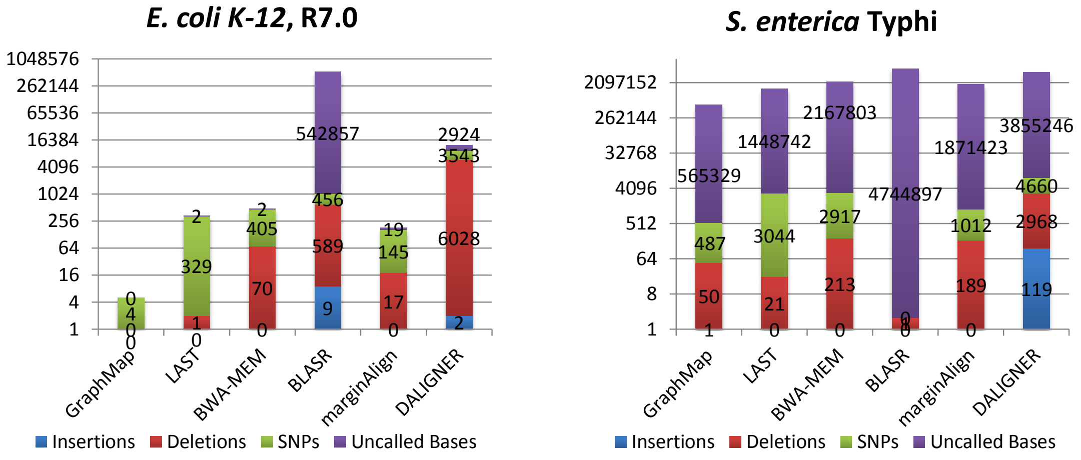

	E. coli **K-12 R7.0**	***S. enterica*** **Typhi**	***E. coli*** **UTI89**	***A. baylyi*** **ADP1**	***B. fragilis*** **BE1**
**GraphMap**	**97%, 135**	**97%, 32**	**99%,** 8	**97%, 54**	**99%,** 25
**LAST**	66%, 117	58%, 26	85%, **9**	60%, 48	91%, **29**
**BWA-MEM**	64%, 90	51%, 21	76%, 7	55%, 37	91%, 23
**BLASR**	27%, 26	19%, 7	36%, 3	32%, 11	53%, 5
**marginAlign**	66%, 97	58%, 22	86%, 8	60%, 37	91%, 22
**DALIGNER**	19%, 56	20%, 14	39%, 5	22%, 23	59%, 26

Note that in the case of the *S. enterica* Typhi dataset, some of the observed variants (typically a few hundred SNPs and a handful of indels) could be true variants from the *S. enterica* Typhi Ty2 strain that was used as reference. Percentage of bases mapped (B%) and average coverage (C) of the genome is reported in the table below (in the format: B%, C; maximum values in each column are bolded).

Supplementary Figure 3. Error rate distributions estimated using different aligners for ONT data

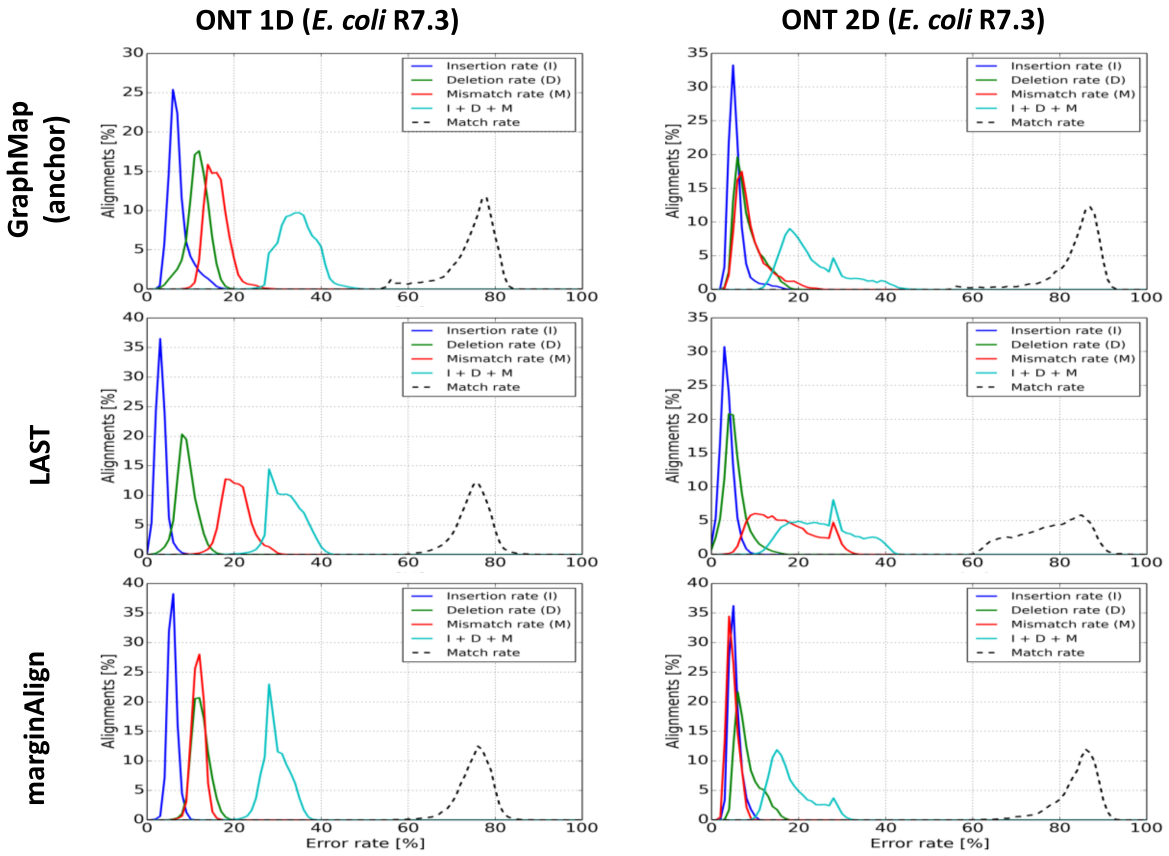

Supplementary Figure 4. Mapping of targeted sequencing reads from Ammar et al.

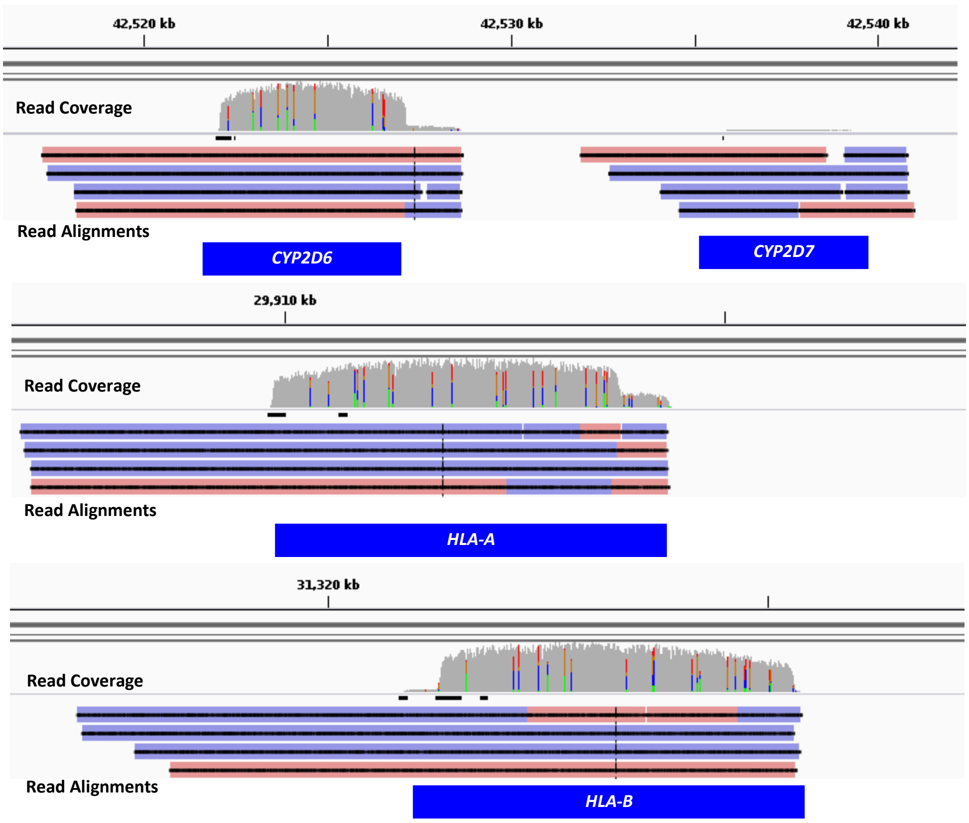

***CYP2D6* (chr22)**

# of on-target reads

GraphMap: 6879
BWA-MEM: 5900
LAST: 5032
BLASR: 2284
marginAlign: 4683
DALIGNER: 1451

***HLA-A* (chr6)**

# of on-target reads

GraphMap: 5176
BWA-MEM: 4400
LAST: 3705
BLASR: 2096
marginAlign: 2703
DALIGNER: 818

***HLA-B* (chr6)**

# of on-target reads

GraphMap: 9032
BWA-MEM: 7567
LAST: 6237
BLASR: 3478
marginAlign: 4767
DALIGNER: 1183

Figures show IGV browser views of GraphMap mappings to the targeted regions. Note that *CYP2D6* has an orthologous gene *CYP2D7* that is adjacent to it with 94% identity and yet has very few reads mapped to it.

Supplementary Table 1. Precision and recall of alignment for GraphMap using various read alignment settings

	Myers bit vector (default)	Gotoh	Anchored Alignment
N. meningitidis	79/79; 73/73	82/82; 75/73	80/79; 73/72
E. coli	80/80; 74/74	83/83; 76/76	80/80; 74/73
S. cerevisiae	77/77; 70/70	80/80; 72/72	79/77; 72/70
C. elegans	78/78; 68/68	81/81; 70/70	78/77; 71/67
***H. sapiens* chr 3**	78/78; 71/71	81/81; 73/73	78/77; 71/70

Results are reported in the format: precision-for-2D-reads/recall-for-2D-reads; precision-for-1D-reads/recall-for-1D-reads.

Supplementary Table 2. Scalability as a function of read length and error rate

CPU time [s]					
			Average read length		
**Error rate**	**1000bp**	**2000bp**	**3000bp**	**4000bp**	**5000bp**
**0.05**	130.7	210.7	278.5	349.5	457.9
**0.10**	125.1	196.5	273.6	358.3	454
**0.15**	119.9	195.9	257	365.1	461.4
**0.20**	114.8	199.3	270.5	348.8	460.1
**0.25**	108.7	196.7	271.9	358.1	485.2

Memory [MB]					
			Average read length		
**Error rate**	**1000bp**	**2000bp**	**3000bp**	**4000bp**	**5000bp**
**0.05**	952	960	972	992	1006
**0.10**	951	960	972	990	1006
**0.15**	951	959	972	989	1011
**0.20**	951	960	972	991	1008
**0.25**	951	960	972	991	1012

As expected, GraphMap's runtime scales roughly linearly with read length and is relatively stable with changes in error rate (*S. cerevisiae* genome). Memory requirements were also found to be stable with varying error rates and increased slightly with read length.

Supplementary Table 3. Testing for reference bias in GraphMap alignments

	SNP Errors (per Mbp)	Insertion Errors (per Mbp)	Deletion Errors (per Mbp)
**BLASR**	0.1 (0.02/0.1)	3.1 (0.04/3.1)	4.0 (0.3/3.7)
**BWA-MEM**	0.2 (0.1/0.1)	2.5 (0.04/2.5)	5.3 (1.7/3.6)
**DALIGNER**	0.4 (0.3/0.1)	1.2 (0.04/1.1)	6.9 (4.1/2.7)
**GraphMap**	0.2 (0.03/0.1)	3.3 (0.05/3.2)	4.1 (0.3/3.8)
**LAST**	1.7 (1.5/0.2)	3.9 (0.05/3.8)	4.6 (0.2/4.4)
**marginAlign**	0.1 (0.03/0.1)	2.0 (0.02/2.0)	4.4 (1.4/3.0)

E. coli K-12 MG1655 reads from Loman *et al.* were mapped to a mutated reference containing 4516 SNPs, 26,961 insertions and 27,133 deletions (see **Methods**). The resulting consensus sequence for each method was compared to the original reference to identify SNP, insertion and deletion errors. The number of errors for each method was normalized by the number of called bases to make them comparable. Errors are reported in the format: Total (# in non-mutated positions/# in mutated positions).

Supplementary Table 4. Speed comparison across mappers on real datasets

	Lambda phage	*E. coli R7.3*	*E. coli* R7.0	*E. coli* UTI89	*S. enterica* Typhi
**GraphMap**	65	49	44	80	44
**LAST**	71	114	112	134	110
**BWA-MEM**	28	32	29	39	37
**BLASR**	2	20	14	41	18
**marginAlign**	0.4	1	2	0.4	0.7
**DALIGNER**	20	6	9	8	3

Results are reported in terms of kilobases mapped per second to account for the wide variation in the number of bases aligned by different mappers.

Supplementary Table 5. Parameters used for generating simulated ONT reads

	2D reads	1D reads
**Accuracy mean**	0.69	0.59
**Accuracy std**	0.09	0.05
**Accuracy min**	0.40	0.40
**Length mean**	5600	4400
**Length std**	3500	3900
**Length min**	100	50
**Length max**	100000	100000
**Error types ratio (mismatch:insertion:deletion)**	55:17:28	51:11:38

Parameters were estimated using LAST alignments with *E. coli* K-12 R7.3 data.

Supplementary Note 1: Evaluating GraphMap on synthetic datasets

On synthetic datasets emulating error profiles from Illumina and PacBio sequencing, we noted that GraphMap and BLAST have high precision and recall (~98%) for both location and alignment measures and are almost indistinguishable in these metrics (**Supplementary Figure 1a**). The slight variations in performance that were observed were not defined by the size of the genomes that were studied. In addition, despite the marked differences in error profiles for Illumina and PacBio, the observed performance metrics were comparable, highlighting the robustness of GraphMap and its similarity to the gold-standard BLAST. Other mappers (BWA-MEM, LAST, DALIGNER and BLASR) exhibit similarly consistent results on Illumina data and PacBio data, with the exception of BLASR being slightly worse on PacBio data (by up to 10% for the human genome). BLASR's results could be a result of it being tuned to specific features of PacBio data that are not adequately captured in our simulation.

Supplementary Note 2: GraphMap's sensitivity on ONT datasets

GraphMap and other mappers (BWA-MEM, LAST, DALIGNER and BLASR) were evaluated on a range of publicly available ONT datasets for their performance (runtime, memory usage) and sensitivity for read mapping. Across all datasets, GraphMap was able to map the most reads and bases, typically mapping more than 95% of the bases and 85% of the reads in a dataset (**Fig. 3b**, **Supplementary Figure 2**, **Supplementary Data 2**). This was despite the exclusion of secondary alignments in GraphMap results and their presence in results for LAST, BWA-MEM and DALIGNER (also used for genome coverage calculations). Overall, LAST was the next best mapper, typically mapping more than 60% of bases (accounting for all secondary alignments; **Supplementary Data 2**). The use of marginAlign with LAST did not improve its sensitivity significantly for these datasets. BWA-MEM results were frequently comparable to that of LAST while DALIGNER and BLASR had lower sensitivity in several datasets (**Supplementary Data 2**). Two of the datasets (*E. coli* UTI89 and *B. fragilis* BE1) contain only high quality 2D reads and associated 1D reads, and thus they only test mappers on a small, high-quality subset of the data. GraphMap was seen to provide a 10-15% increase in sensitivity for such reads. On the full datasets, GraphMap typically provided a 50% improvement in mapped bases compared to LAST. The datasets *A. baylyi* ADP1[1] and *B. fragilis* BE1[2] were recently published and provide a more current perspective on GraphMap's utility for all data and high-quality 2D data, respectively. On a recent MinION MkI dataset (*E. coli* MAP006-1), GraphMap provided an 18% improvement in mapped bases compared to other mappers (**Supplementary Data 2**).

Supplementary references

1. Madoui, M.-A. *et al.* Genome assembly using Nanopore-guided long and error-free DNA reads. *BMC Genomics* **16**, 327 (2015).
2. Risse, J. *et al.* A single chromosome assembly of Bacteroides fragilis strain BE1 from Illumina and MinION nanopore sequencing data. *bioRxiv* (2015).
